# Prevalence of purging at age 16 and associations with negative outcomes among girls in three community-based cohorts

**DOI:** 10.1111/jcpp.12283

**Published:** 2014-06-27

**Authors:** Francesca Solmi, Kendrin R Sonneville, Abigail Easter, Nicholas J Horton, Ross D Crosby, Janet Treasure, Alina Rodriguez, Marjo-Riitta Jarvelin, Alison E Field, Nadia Micali

**Affiliations:** 1 Behavioural & Brain Sciences Unit, Institute of Child Health, University College LondonLondon, UK; 2 Division of Adolescence Medicine, Boston's Children Hospital, Harvard Medical SchoolBoston, MA, USA; 3 Department of Mathematics & Statistics, Amherst CollegeAmherst, MA, USA; 4 Department of Neuroscience, Neuropsychiatric Research Institute, University of North DakotaFargo, ND, USA; 5 Eating Disorder Unit, Institute of Psychiatry, King's College LondonLondon, UK; 6 Department of Epidemiology and Biostatistics, School of Public Health, Imperial College LondonLondon, UK; 7 Mid Sweden University, Department of Psychology, Campus ÖstersundÖstersund, Sweden; 8 Institute of Health Sciences, University of OuluOulu, Finland; 9 Biocenter Oulu, University of OuluOulu, Finland; 10 Unit of Primary Care, Oulu University Hospital, OYSOulu, Finland; 11 Department of Children and Young People and Families, National Institute for Health and WelfareOulu, Finland

**Keywords:** Adolescence, epidemiology, prevalence, eating behaviour, eating disorder

## Abstract

**Background:**

The comorbidity of purging behaviours, such as vomiting, inappropriate use of laxatives, diuretics or slimming medications, has been examined in literature. However, most studies do not include adolescents, individuals who purge in the absence of binge eating, or those purging at subclinical frequency. This study examines the prevalence of purging among 16-year-old girls across three countries and their association with substance use and psychological comorbidity.

**Methods:**

Data were obtained by questionnaire in 3 population-based cohorts (Avon Longitudinal Study of Parents and Children (ALSPAC), United Kingdom, *n* = 1,608; Growing Up Today Study (GUTS), USA, *n* = 3,504; North Finland Birth Cohort (NFBC85/86), Finland, *n* = 2,306). Multivariate logistic regressions were employed to estimate associations between purging and outcomes. Four models were fit adjusting for binge eating and potential confounders of these associations.

**Results:**

In ALSPAC, 9.7% of girls reported purging in the 12-months prior to assessment, 7.3% in GUTS, and 3.5% in NFBC. In all 3 cohorts, purging was associated with adverse outcomes such as binge drinking (ALSPAC: odds ratio (OR) = 2.0, 95% confidence interval (CI) = 1.4–2.9; GUTS: OR = 2.5, 95% CI = 1.5–4.0; NFBC: OR = 1.7, 95% CI = 1.0–2.8), drug use (ALSPAC: OR = 2.9, 95% CI = 1.8–4.7; GUTS: OR = 4.5, 95% CI = 2.8–7.3; NFBC: OR = 4.1, 95% CI = 2.6–6.6), depressive symptoms in ALSPAC (OR = 2.2, 95% CI = 1.5–3.1) and GUTS(OR = 3.7, 95% CI = 2.2–6.3), and several psychopathology measures including clinical anxiety/depression in NFBC (OR = 11.2, 95% CI = 3.9, 31.7).

**Conclusions:**

Results show a higher prevalence of purging behaviours among girls in the United Kingdom compared to those in the United States and Finland. Our findings support evidence highlighting that purging in adolescence is associated with negative outcomes, independent of its frequency and binge eating.

## Introduction

Purging behaviours are described as the inappropriate use of laxatives, diuretics and slimming medications, as well as self-induced vomiting to control weight. Purging commonly cooccurs with other eating disordered behaviours and cognitions, as described in diagnostic definitions of Anorexia Nervosa Binge-Purge (AN-BP) or Bulimia Nervosa (BN) (American Psychiatric Association, 2013). However, evidence suggests that some individuals engage in purging, but do not have AN nor do they engage in binge eating. It has been suggested that these individuals have ‘purging disorder’ (PD) (Keel, Haedt, & Edler, 2005). However, due to the paucity of studies on PD it is not an official eating disorder (ED) in the Diagnostic and Statistical Manual for Mental Disorders version 5 (DSM-5), but it rather remains in the heterogeneous group of other specified feeding and eating disorders (OSFED) (American Psychiatric Association, 2013). This appears to underplay evidence on the increasing prevalence of purging behaviours in both clinical and nonclinical populations and on their associated comorbidity (Ackard, Cronemeyer, Franzen, Richter, & Norstrom, 2011; Field et al., 2012).

Several studies (Abebe, Lien, Torgersen, & von Soest, 2012; Allen, Byrne, Oddy, & Crosby, 2013; Fink, Smith, Gordon, Holm-Denoma, & Joiner, 2009; Haedt & Keel, 2010; Keel et al., 2005; Keel, Holm-Denoma, & Crosby, 2011; Keel, Wolfe, Gravener, & Jimerson, 2008; Spoor, Stice, Burton, & Bohon, 2007; Stice, Marti, & Rohde, 2013; Stice, Marti, Shaw, & Jaconis, 2009) have investigated its clinical relevance and the differences between individuals with PD and both individuals with BN-P and controls, with compelling results. Compared to healthy women, those who only purge have a higher prevalence of depression (Keel et al., 2005, 2008; Wade, 2007), anxiety (Keel et al., 2005, 2008), impulsivity (Fink et al., 2009), impaired psychosocial functioning (Haedt & Keel, 2010; Spoor et al., 2007), alcohol consumption (Abebe et al., 2012; Anderson, Martens, & Cimini, 2005; Field et al., 2012), general Axis I & II psychopathology (Keel et al., 2005), and drive for thinness and body dissatisfaction (Fink et al., 2009).

While shedding light on the comorbidity of purging behaviours, these studies share theoretical and methodological similarities. First, most studies (Fink et al., 2009; Haedt & Keel, 2010; Keel et al., 2005, 2008; Spoor et al., 2007; Wade, 2007) set a frequency threshold of at least one or two purging episodes per week as the criterion to identify purgers. However, these cut-offs are not empirically derived and, while helping understand more clinically relevant cases, it is important to assess these associations over the full range of prevalences occurring at the population level. Second, the use of adult samples is common in the literature on purging and PD. However, several studies suggest that, although the prevalence of purging behaviours remains high through early young adulthood (Neumark-Sztainer, Wall, Larson, Eisenberg, & Loth, 2011), that of PD peaks in mid/late adolescence (Abebe et al., 2012; Field et al., 2012; Stice et al., 2013, 2009). Moreover, a recent study found that purging, compared to other ED behaviours, was associated with the most severe comorbidity (i.e. anxiety, depression, alcohol consumption and high scores in the EAT-12 and BITE-30 ED scales) in a sample of 14 to 23 year olds (Abebe et al., 2012). Focusing on adult populations could thus miss the more severe behaviours and psychopathology typically seen in adolescents.

This study investigates the prevalence and correlates of purging behaviours, regardless of their frequency, among adolescents using population samples based in different countries. The primary aim of this study was to explore the prevalence of purging behaviours and their association with adverse outcomes in 16 year-old girls across 3 general population samples in the United Kingdom, United States, and Finland. The rationale for choosing 16 year-old girls was the high prevalence of purging behaviours, suggested by literature, in this group. Boys were not included in this study based on the a priori knowledge of their lower use of purging methods (Abebe et al., 2012; Field, Camargo, Taylor, Berkey, Frazier, et al., 1999) and, therefore, on considerations of statistical power of the analyses. The secondary aim was to investigate whether the prevalence of purging behaviours varies across countries, which could suggest a role of cultural factors in influencing purging behaviours.

## Methods

### Samples

This study employed data from three general population cohort studies.

The Avon Longitudinal Study of Parents and Children (ALSPAC) is a longitudinal study of women and their children. All pregnant women expected to deliver between 1^st^ April 1991 and 31^st^ April 1992 were invited to participate in the study. Participants' informed written consent was obtained prior to recruitment and the study was approved by the ALSPAC Law and ethics committee. More details on the study are given elsewhere (Boyd et al., 2012). At age 16, 10,388 adolescents were sent a postal questionnaire.

The Growing Up Today Study (GUTS) began in 1996 by recruiting mothers participating in the ongoing Nurses' Health Study II (NHS) who had children aged 9–14 (Field, Camargo, Taylor, Berkey, & Colditz, 1999). The aims of the project were to study the diet, activity and weight change in their offspring during adolescence. Parental informed consent was sought prior to sending the first questionnaire and invitation letter. Approximately 68% of the invited participants (*n* = 9,039) returned completed questionnaires, thereby assenting to participate in the study. Participants were sent questionnaires annually from 1996 to 2001, and biennially from 2003 to 2011. Survey waves 1998, 1999, 2000, 2001 and 2003 correspond to the years GUTS participants were age 16. Approximately 6,755 of the 9,039 participants were eligible for this study because they were aged 16 during one of these survey waves. GUTS was approved by the Human Subjects Committee at Brigham and Women's Hospital.

The Northern Finland Birth Cohort 1986 (NFBC86, hereafter referred to as NFBC) is a longitudinal study that recruited all pregnant women expecting to deliver between 1^st^ July 1985 and 30^th^ June 1986 in the provinces of Oulu and Lapland. Baseline data were supplemented by data collected with postal questionnaires at age 15/16, hospital records and registry data. At age 16, 9,215 adolescents and parents were sent a postal questionnaire (Kantomaa et al., 2013; Kantomaa, Tammelin, Demakakos, Ebeling, & Taanila, 2010). The ethics committee of Northern Ostrobotnia Hospital District approved the study, and both parents and adolescents gave written informed consent.

### Measures

#### Purging behaviours

ALSPAC and GUTS assessed eating and weight control behaviours using a set of questions adapted from the Youth Risk Behaviour Surveillance System (YRBSS) questionnaire (Kann et al., 1996). These questions have been validated among girls, with relatively high specificity and negative predictive values of self-reported purging and binge eating (Field, Taylor, Celio, & Colditz, 2004). Participants were asked how often, in the previous year, they had made themselves vomit or had taken laxatives (or other slimming medications, in ALSPAC) to lose weight or avoid weight-gain. The two variables were combined into a binary variable indicating whether the adolescent had engaged in any purging behaviours or not in the previous year.

In NFBC, adolescents were asked about their use of vomiting, laxatives or other slimming medications to control their weight. Possible answers were ‘never’, ‘occasionally’ and ‘often’. A combined variable was generated indicating whether participants had engaged in any purging behaviours in the previous year.

#### Outcomes

##### Binge drinking

Binge drinking was assessed in ALSPAC using an item of the Alcohol Use Disorders Identification Test (AUDIT), a World Health Organization (WHO) (Babor, Higgins-Biddle, Saunders, & Monteiro, 2001) screening tool developed to screen for excessive drinking, asking how many times the participant has ≥6 units of alcohol in one occasion. Possible answers were dichotomised as ‘never’ or ‘at least monthly’. A question on binge drinking was included in the 1998, 1999, 2000, 2001 and 2003 GUTS questionnaires. Participants were asked about the frequency in the past year of drinking ≥4 drinks over a few hours, which was the frequency threshold to define binge drinking among female participants. Participants were classified as binge drinkers if reporting at least one episode of binge drinking per month. In NFBC, girls were asked the frequency in the past month of consuming >4 drinks in one occasion. Possible answers were coded as ‘never’, or ‘at least weekly’.

##### Drug use

In ALSPAC, adolescents were asked about consumption of: cannabis, cocaine, LSD, ecstasy, amphetamines, mushrooms, heroin, ketamine, crack and steroids. In GUTS, participants were asked, on the 1999, 2001 and 2003 questionnaires, whether they had used marijuana or hashish, cocaine, crack (1999, 2001), heroin, ecstasy, PCP (1999, 2001), GHB (1999, 2001), LSD, mushrooms, ketamine (1999, 2001), Rohypnol (1999, 2001, and 2003) and amphetamines/speed (1999, 2001, 2003). In NFBC, girls were asked if they had used ‘sedatives, sleeping pills, pain killers without alcohol’, ‘alcohol and pills together’ and ‘ecstasy, heroin, cocaine, amphetamines LSD or other similar drugs’. In all three samples, a dichotomous variable was created indicating any use of each drug, regardless of frequency. Subsequently, all variables were added together and a binary variable was derived indicating whether the adolescent had used any drugs in the previous year (e.g.: score ‘0’ = no use of drugs; score ‘≥1’ = use of at least one drug). Cannabis was not grouped with other drugs on the a priori knowledge of its association with overeating (Field et al., 2012).

##### Smoking

In all three cohorts smoking was recorded as binary variable indicating whether adolescents were smoking currently (NFBC) or in the 12 months prior to assessment (ALSPAC, GUTS).

##### Psychopathology

The Short Moods and Feelings Questionnaire (Messner et al., 1995) (SMFQ), a 13-item questionnaire developed as a screening tool to detect symptoms of depressive disorders in children and adolescents aged 6–17, was used to measure depressive symptoms in ALSPAC. Each item in the questionnaire is scored on 0-2 a scale (0 = true, 1 = sometimes true, 2 = true). A cut-off score of 8 was used to identify clinically depressive states, as shown in previous literature (Kuo, Stoep, & Stewart, 2005; Messner et al., 1995). The SMFQ has good internal construct validity in both clinical (Messner et al., 1995) and general population samples (Sharp, Goodyer, & Croudace, 2006). In 1999, 2001 and 2003, depressive symptoms were assessed using the six-item validated scale of the McKnight Risk Factor Survey (MRFS) IV (Shisslak et al., 1999) in GUTS. Scores in the top decile were considered as indicating depressive symptoms. In NFBC, the ‘problems’ section of the Youth Self-Report (Achenbach, 1991) (YSR) questionnaire was used to identify the presence of internalising (anxious/depressed; withdrawn; somatic complaints; thoughts problems; attention problems) and externalising (social problems; rule-breaking behaviour; aggressive behaviours) behaviours. The overall score for each subscale was recorded into a 3-level ordinal variable using previously employed cut-offs of circa 84^th^ and 90^th^ percentile indicating: normal, subclinical and clinical ranges (Kantomaa et al., 2010).

#### Covariates

##### Binge eating

Binge eating was assessed with a 2-part question in ALSPAC and GUTS. Participants were first asked about the frequency during the past year of eating a very large amount of food. Girls reporting overeating were directed to a follow-up question asking whether they felt loss of control (LOC) during these episodes, such as they could not stop eating even if they wanted. Binge eating was defined as eating a very large amount of food in a short amount of time at least monthly and feeling out of control during the eating episode. In NFBC, adolescents were asked about the frequency of eating a large amount of food in a short period of time. A binary variable was created separating participants who had answered that they had ‘never’, ‘hardly ever’ or ‘occasionally’ binged from those who had reported doing so ‘once a month’, ‘once a week’, ‘2/3 times per week’ and ‘daily’(Khalife et al., 2014). This choice was justified by the need to account for the absence of a measure for LOC, essential in defining binge eating. This classification was aimed at discriminating more common episodes of overeating from real binges.

##### Body mass index (BMI)

In ALSPAC, BMI (kg/m²) was obtained from objective weight and height measurements. In GUTS, BMI was calculated using self-reported weight and height, as this has been found to be a valid measure in large epidemiological studies (Fonseca et al., 2010). In NFBC, BMI was calculated from objective examinations of weight and height measurements (*n* = 3,290) and self-reported measurement for those girls not participating in the examination (*n* = 423). The correlation between BMI derived from measured and self-reported data were *r* = .7. In all three studies, BMI was used as a continuous variable.

##### Sociodemographic

In ALSPAC, information on maternal marital status and education was collected at enrolment and dichotomised as ‘married/cohabiting’ or ‘single parent’, and as ‘O-level or equivalent’(obtained at 16 years) or ‘A-levels (obtained at 18 years) or above’(secondary school level exams and University degree), respectively. Maternal education was not collected in GUTS participants because the study was nested in the Nurses’ Health Study and therefore all women had a similar level and type of education. In 2001, mothers of GUTS participants self-reported their marital status, which was dichotomised as ‘currently married’ versus ‘widowed, divorced, separated, and never married’. In NFBC, family structure and maternal education were obtained from parents at the 16-year follow-up and coded as either ‘married/cohabiting’ or ‘single parent’; and ‘basic compulsory education’ versus ‘upper secondary education or above’ (secondary school exams and university degrees).

### Data analyses

For each study, univariate and multivariate logistic and multinomial logistic regressions were used to calculate odds ratios (ORs) and 95% confidence intervals (CIs) for the association between purging behaviours and the outcomes under study, and the potential confounding role of a number of covariates. After fitting a univariate model for the association between each outcome and the exposure (purging behaviours), three additional models were fit, adjusting for: (a) binge eating; (b) 1+ age, BMI, maternal education and marital status in ALSPAC and NFBC, and marital status only in GUTS; (c) 2+ binge drinking adjusted for smoking and vice-versa given the high cooccurrence of the two. ALSPAC and NFBC analyses were conducted using Stata12 (StataCorp., 2011). GUTS analyses were conducted using SAS version 9.3 (SAS, 2011).Girls with any missing data on the variables included in the models were excluded and all models were based on complete cases. Prevalence of purging was calculated over the number of complete cases. Differences in prevalence of purging behaviours across the three samples were calculated with a *z*-test for difference in proportions. Differences in sociodemographic characteristics between exposed and unexposed girls were investigated for the subsample of adolescents included in the analyses using cross-tabulations and ANOVA according to the nature of the variable. Girls with complete data on the exposure and outcomes included in the analyses were compared against those who had returned the questionnaire at 16 but had incomplete data. Variables included as covariates in regression models were identified through a priori assumptions of associations with exposure and outcomes based on previous literature. Finally, a meta-analysis was undertaken to compare results on prevalence and comorbidity across the three cohorts and to estimate heterogeneity. Odds ratios and 95% confidence intervals from fully adjusted models were employed in the meta-analyses. A random effects model was employed on the assumption of variability between studies. Given the small sample size, I Squared (*I*^2^) statistics was employed to test for homogeneity between studies. Literature suggests that values of 25%, 50% and 75% represent low, moderate and high heterogeneity (Higgins, Thompson, Deeks, & Altman, 2003).

## Results

### Missing data and attrition

In ALSPAC, 4,462 girls received the questionnaire at 16, 2,742 (61.5%) returned it and 1,608 (36%) had complete information on exposure, outcomes and covariates (Table 1). Lower maternal education (fully observed = 53.8%, some missing = 67.8%, *p* < .0001), having a single mother (fully observed = 17.4%, some missing = 23.5%, *p* < .0001) and higher BMI (fully observed = 21.6, some missing = 21.9, *p* = .05) were associated with having incomplete information on outcomes and exposure.

**Table 1:** Demographic characteristics of the 3 samples

	ALSPAC *N* (%)	GUTS *N* (%)	NFBC *N* (%)
Number of questionnaires returned	2,742 (61.5)	4,614 (68.3)	3,592 (82.1)
Complete cases	1,608 (36.0)	3,504 (52.0)	2,306 (56.7)
Any purging in the previous year^a^	157 (9.7)	255 (7.3)	81 (3.5)
Purging without binge eating	89 (59.7^c^)	192 (75.6^c^)	58 (71.6^c^)
Child's ethnicity			
White	1,539 (96.9)	3393 (96.8)	2,306 (100.0)
Nonwhite	49 (3.1)	111 (3.2)	0 (0.0)
Maternal education^b^			
Up to O-level (ALSPAC); Comprehensive level (NFBC)	821 (51.1)	–	1,499 (65.0)
A-level or more (ALSPAC); Matriculation exam (NFBC)	787 (48.9)	–	807 (35.0)
Maternal marital status			
Single (Single parent/divorced/widowed)	261 (16.2)	378 (10.8)	281 (12.2)
Married or cohabiting	1,347 (83.8)	3,126 (89.2)	2,025 (87.8)

	ALSPAC Mean (*SD*)	GUTS Mean (*SD*)	NFBC Mean (*SD*)
Age (years)	16.7 (0.2)	16.5 (0.3)	15.2 (0.5)
BMI	21.6 (3.5)	21.7 (3.3)	21.1 (3.1)

a Prevalence of purging calculated over the number of complete cases.

b In GUTS, data on maternal education are not presented as the cohort is composed of the offspring of mothers participating in the NHSII (Nurses’ Health Study), therefore mothers, by definition, had the same level of education.

c Proportion of girls who only purge calculated over the number of girls who purge irrespectively of bingeing.

In GUTS, 4,915 of the approximately 6,755 (72.8%) girls in the eligible sample returned a questionnaire at age 16 and, of those, 4,614 (93.9%) responded to questions about purging behaviours (Table 1). For the analyses including binge drinking and cigarette smoking, 3,062 (66.4%) girls had complete information on exposure, outcomes and covariates. Because drug use and depressive symptoms were not assessed in all survey waves, the total sample for these outcomes was 2,579. Of these girls, 1,813 (70.3%) were included in the analyses on marijuana, other drug use, and depressive symptoms as they had complete information on all measures. 3,504 of the 4,614 (75.9%) girls were included in at least one of these analysis samples. In the GUTS sample, individuals with fully observed exposure and outcome data had a higher BMI than individuals with some missing exposure or outcome data (fully observed = 21.7, some missing = 21.5, *p* = .04). No differences were seen by age or maternal marital status.

In NFBC, 4,271 girls were sent the questionnaire at 16, 3,598 returned it and consented to participate and 2,306 (56.7%) had complete information on all variables included in the analyses (Table 1). Having a single mother (fully observed = 13.5%, some missing = 18.7%, *p* = .001) was associated with having incomplete information on outcomes and exposure.

As shown in Table S1 (available online), all three complete case samples were representative of the whole sample of adolescents who were eligible for participating in the age 16 round of data collection and of those who returned the questionnaires on baseline maternal characteristics and ethnicity. Only in ALSPAC maternal education was higher in complete cases compared to the eligible sample and that of those who returned the questionnaire.

### Study participants and purging behaviours

In ALSPAC, girls who purged had higher levels of maternal education (*p* = .007) and BMI (*p* = .01) than those who did not. In GUTS and NFBC, no differences were found between girls who did and did not purge (Table S2).

A total of 157 (9.8%), 255 (7.3%) and 81 (3.5%) girls reported any purging and 89 (59.7%), 192 (75.6%) and 58 (71.6%) of them reported purging without binge eating in the previous year in ALSPAC, GUTS and NFBC, respectively (Table 1). Prevalence of purging was higher in ALSPAC and GUTS compared to NFBC (ALSPAC vs. GUTS, *z* = 3.03, *p* = .002; ALSPAC vs. NFBC, *z* = 8.05, *p* = .0002; GUTS vs. NFBC, *z* = 6.01, *p* = .0002).

**Figure 1:**
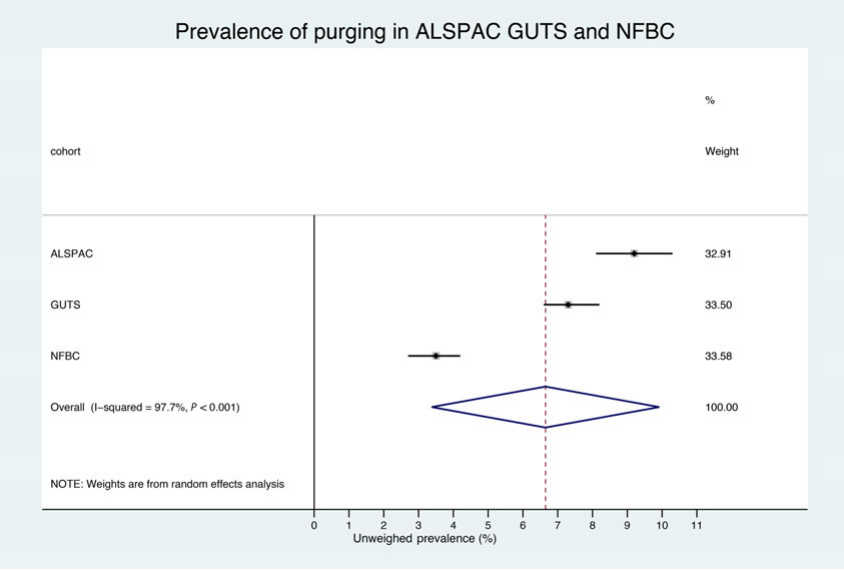
Meta-analysis of the prevalence of purging behaviours in ALSPAC, GUTS, and NFBC

The meta-analysis (Figure1) showed a pooled prevalence of 6.6% (95% CI: 3.3–9.9; *p* < .0001). Strong evidence of great heterogeneity between the studies was found (*χ*² = 85.3, *p* < .0001; *I*² = 97.7%).

### Associations between purging and studied outcomes

In univariate models, and in models adjusting for any binge eating episodes, purging was associated with high odds of reporting all outcomes measured in all three samples (Table 2). After adjusting for sociodemographic variables, purging was still associated with high odds of binge drinking, smoking, cannabis use and any other drug use in ALSPAC, GUTS and NFBC, although purging was not associated with binge drinking less than weekly in NFBC (Table 2). In multivariate models, purging was associated with high odds of having depressive symptoms in ALSPAC and GUTS and clinical levels of anxiety, somatic, attention problems and rule-breaking behaviours in NFBC (Table 3).

**Table 2:** Crude and adjusted Odds Ratios (OR & 95% CI) for the association between purging behaviours and the risk for binge drinking, using drugs, and smoking

Cohort	Crude OR (95% CIs)	Adjusted^a^ OR (95% CIs)	Adjusted^b^ OR (95% CIs)	Adjusted^c^ OR (95% CIs)
ALSPAC (*n* = 1,608)				
Binge drinking (monthly or more)	3.0 (2.2–4.2)**	2.9 (2.0–4.3)**	2.9 (2.0–4.1)**	2.0 (1.4–2.9)**
Any smoking (since age 15)	3.6 (2.5–5.2)**	3.4 (2.3–5)**	3.4 (2.3–5.1)**	2.7 (1.8–4.0)**
Cannabis use (previous year)	3.2 (2.3–4.5)**	2.9 (2.1–4.2)**	2.9 (2.1–4.2)**	–
Drug use (Used one or more since age 15)	3.3 (2.1–5.1)**	2.8 (1.8–4.5)**	2.9 (1.8–4.7)**	–
GUTS				
Binge drinking (monthly or more) (*n* = 3,062)	3.3 (2.1–5.2)**	2.7 (1.7–4.3)**	2.7 (1.7–4.4)**	2.5 (1.5–4.0)**
Any smoking (previous year) (*n* = 3,062)	1.9 (1.4–2.5)**	1.9 (1.4–2.6)**	1.9 (1.4–2.6)**	1.7 (1.2–2.3)**
Cannabis use (previous year) (*n* = 1813)	3.9 (2.5–5.9)**	3.3 (2.1–5.1)**	3.5 (2.2–5.4)**	–
Any drug use (previous year) (*n* = 1813)	4.9 (3.1–7.6)**	4.5 (2.8–7.2)**	4.5 (2.8–7.3)**	–
NFBC (*n* = 2,306)				
Binge drinking (weekly or more)	2.9 (1.9–4.7)**	2.7 (1.7–4.3)**	2.7 (1.7-4.3)**	1.7 (1.0-2.8)**
Any current smoking	4.3 (2.6–6.3)**	3.8 (2.4–6.1)**	3.8 (2.4-6.1)**	3.0 (1.8-5.0)**
Cannabis use (previous year)	5.2 (2.9–9.1)**	4.6 (2.6–8.1)**	4.5 (2.5-7.9)**	–
Any drug use (previous year)	4.8 (3.1–7.6)**	4.1 (2.6–6.7)**	4.1 (2.6-6.6)**	–

*p ≤ .05; **p ≤ .01.

a Adjusted for binge eating.

b Adjusted for (i) binge eating, age, BMI, maternal education, maternal marital status (ALSPAC, NFBC); (ii) binge eating, age, BMI, maternal marital status (GUTS).

c Binge drinking analysis in addition adjusted for smoking; smoking analysis in addition adjusted for binge drinking.

**Table 3:** Odds Ratios (OR & 95% CI) of the association between purging behaviours and psychiatric comorbidity

Cohort	Crude OR (95% CIs)	Adjusted^a^ OR (95% CIs)	Adjusted^b^ OR (95% CIs)
ALSPAC (*n* = 1,608)			
Depressed mood (previous month)	2.9 (2.1–4.1)**	2.2 (1.5–3.1)**	2.2 (1.5–3.1)**
GUTS (*n* = 1,813)			
High depressive symptoms	4.7 (3.0–7.5)**	3.6 (2.1–6.0)**	3.7 (2.2–6.3)**
NFBC (*n* = 2,306)			
Anxious/depressed			
Subclinical	7.9 (4–15.5)**	6.4 (3.2–12.9)**	6.3 (3.1–12.7)**
Clinical	14.7 (5.4–39.4)**	10.5 (3.7–29.7)**	11.2 (3.9–31.7)**
Withdrawn			
Subclinical	8 (3.4–19.2)**	6.8 (2.7–16.7)**	6.5 (2.6–16.1)**
Clinical	3.7 (0.5–30.1)**	2.1 (0.2–17.5)	2.1 (0.2–18.1)
Somatic			
Subclinical	4.7 (2.5–8.8)**	3.7 (1.9–7.1)**	3.6 (1.9–6.9)**
Clinical	26 (8.7–77.2)**	19.4 (6.2–60.6)**	20.1 (6.5–65.3)**
Social problems			
Subclinical	1.5 (0.4–6.5)	1.4 (0.3–6.1)	1.4 (0.3–5.9)
Clinical	–		
Thought problems			
Subclinical	1.8 (0.6–5.2)	1.6 (0.8–5.1)	1.6 (0.6–4.5)
Clinical	3.4 (0.7–14.9)**	2.2 (0.4–10.1)	2.4 (0.5–11.2)
Attention problems			
Subclinical	2.6 (1.2–5.6)*	2.1 (0.9–4.6)	2.1 (0.9–4.6)
Clinical	18.2 (4.3–77.8)**	18.8 (4.2–84.2)**	19.4 (4.3–88.2)**
Rule-breaking behaviour			
Subclinical	6.7 (4.1–11.3)**	5.9 (3.5–10.1)**	5.9 (3.5–10.1)**
Clinical	15.2 (7.6–30.5)**	12.5 (6.1–25.6)**	12.1 (5.9–24.9)**
Aggressiveness			
Subclinical	3.7 (1.6–8.5)**	2.8 (1.2–6.7)*	2.8 (1.2–6.7)*
Clinical	4.4 (1.5–12.7)**	2.6 (0.8–7.9)	2.5 (0.8–7.9)

* *p* ≤ .05;

** *p* ≤ 0.01.

a Adjusted for binge eating.

b Adjusted for (i) binge eating, age, BMI, maternal education, maternal marital status (ALSPAC, NFBC); (ii) binge eating, age, BMI, maternal marital status (GUTS).

Pooled ORs from meta-analysis for binge drinking, cigarette smoking, drug use, cannabis use and depression were 2.1 (95% CI: 1.6–2.7, *p* < .0001), 2.3 (95% CI: 1.6–3.3, *p* < .0001), 3.8 (95% CI: 2.8–4.9, *p* < .0001), 3.4 (95% CI: 2.6–4.3) and 3.8 (95% CI: 1.8–8.2, *p*: .001) respectively. Evidence of moderate heterogeneity was observed for depression and cigarette smoking, but not for any of the other outcomes (Figures S1–S5).

## Discussion

This is the first study looking at associations between purging behaviours, irrespective of their frequency, and several negative outcomes (smoking, binge drinking, drug use, psychopathology) across three population cohorts of adolescents.

The results from these three samples share important similarities and some differences. The prevalence of purging was high across the three cohorts (9.8% ALSPAC, 7.3% in GUTS, 3.5% in NFBC), although higher in the United Kingdom than in the United States and the Finnish ones (ALSPAC > GUTS > NFBC), (it is of note that GUTS did not ask about slimming pills, which could account for some of the difference seen with ALSPAC). We also found strong evidence of heterogeneity between the studies (*I*^2^ = 97%). This finding could be explained by the presence of cultural differences across the three countries and more evidence is needed to evaluate this hypothesis. A recent North American study found around 13% of their sample reporting purging behaviours in midadolescence (Neumark-Sztainer et al., 2011) suggesting that differences seen between the ALSPAC and GUTS cohort in this study could be due to socioeconomic homogeneity of the American sample and not to actual differences, and that secular trends could account for the higher prevalence of purging seen in this more recent study. Data for all cohorts were collected in the early 2000s, therefore an effect of time on these results seems unlikely. Mean age in NFBC was 15.2 years compared to 16.7 years in ALSPAC and 16.5 years in GUTS, which could suggest older age of onset of purging behaviours, as previously shown (Field et al., 2012; Stice et al., 2009). In ALSPAC, GUTS and NFBC, 59.7%, 75.6% and 71.6%, respectively, of the girls reporting purging did not report binge eating. Compared to previous studies using diagnostic thresholds (Abebe et al., 2012; Allen et al., 2013; Field et al., 2012) we found higher prevalence of purging, although our results are comparable to those of other studies investigating prevalence of any purging behaviours (Neumark-Sztainer et al., 2011).

As documented in previous studies (Fink et al., 2009; Keel et al., 2005, 2008; Spoor et al., 2007; Wade, 2007), we found purging behaviours associated with a number of negative outcomes. In all three cohorts, purging was associated with smoking, binge drinking, cannabis and other drugs use. In ALSPAC and GUTS, purging was associated with depressive symptoms, and in NFBC with a number of internalising and externalising behaviours. However, whereas previous studies used frequency thresholds to define purging, this study found these associations present irrespectively of the frequency with which the subjects purged. Two studies had formerly observed higher Axis I comorbidity among adolescents who binge eat and purge than in those who do not engage in either behaviours or purged only (Fink et al., 2009; Keel et al., 2008). Our study suggests that purging alone is associated with substantial comorbidity. Most of the girls who purged did not binge eat and adjusting for binge eating did not alter the association between purging and the outcomes investigated.

These results should be interpreted in the light of some limitations. First, data were analysed cross-sectionally and, therefore, the developmental trajectory cannot be inferred. Longitudinal analyses could have provided better estimates of changes in prevalence and prospective risk factors. However, not all three cohorts had collected data throughout adolescence with the same frequency thus limiting the scope for longitudinal comparisons. Regardless of temporality, however, our findings reveal a clustering of risk behaviours among adolescents, which may have important implications for prevention and intervention.

The information on purging was gathered by self-report questionnaire; however, ALSPAC and GUTS measures have been validated with excellent specificity and negative predictive value.

In all three cohorts ethnic and socioeconomic diversity is under-represented. While NFBC is representative of the Finnish population, ALSPAC and GUTS are not representative of the UK and US populations. Inferences on the generalisability of these results to adolescents from different ethnic backgrounds or lower socioeconomic statuses should thus be made with caution. Information on maternal marital status was only available at birth in ALSPAC, which did not allow testing the effect of marital status concurrent or closer on the studied outcome. However, adjusting for marital status did not affect the direction, strength and size of the association in GUTS and NFBC, suggesting that this result could be generalised to ALSPAC as well.

Finally, different measures were used across the cohorts. However, we focused on any purging in the previous year as the main exposure and outcomes were recoded as to increase their comparability. The similarity of results observed despite measurement differences, suggests the presence of commonalities proper of exposed individuals regardless of differences in measurements.

Despite these limitations, this study has important strengths. It employed three large population-based cohorts, with several advantages. It is known that the minority of people with an ED receive treatment (Swanson, Crow, Le Grange, Swendsen, & Merikangas, 2011), thus the generalisability of results from clinical samples is questionable. In population-based cohorts (where behaviours and not full-diagnoses are used) less severe cases are likely to be included in the sample. This can attenuate results since the minority of ‘cases’ will meet clinical thresholds. The strong associations we observed add to the evidence that even low-frequency and low-level purging behaviours (among individuals that might not present to services) in adolescence might have negative consequences. Disordered eating behaviours are known to appear in adolescence and early adulthood. This study is an important first step in investigating the prevalence of early symptoms and their correlates, Future longitudinal research should aim at investigating whether individuals experiencing disordered eating behaviours are more likely to develop full-scale diagnoses or adverse consequences across a range of psychological, behavioural and social domains. Secondly, our samples were larger than those employed by previous studies, thus increasing power of analyses and reducing the role of chance.

## Conclusions

These findings have several important implications. Firstly, they stress the clinical relevance of purging and the necessity of including PD in future diagnostic manuals, since its inclusion in DSM-5 as a separate diagnosis did not occur. Secondly, they suggest that at a population level individuals who purge are likely to have a series of concurrent risk-taking and psychopathological behaviours. While rather ample literature exists on substance abuse and addiction in BN (O'Brien & Vincent, 2003), recent findings suggest that adolescents who purge are those at highest risk of having this type of comorbidity (Abebe et al., 2012). Our results on the association between sporadic purging and substance use hints to an underlying trait common to all behaviours. The role of impulsivity has been widely investigated in relation to BN (Favaro et al., 2004; Fischer, Smith, & Anderson, 2003); however, the same research has not been undertaken among individuals who purge only (Fink et al., 2009). Moreover, our results on the association between purging behaviours in adolescents girls and attentions problems in NFBC, also echo those of a recent study finding that only children at high risk of developing purging-type ED due to being born to mothers with lifetime purging behaviours showed poorer performance in a task measuring sustained attention, the primary neurocognitive deficit of ADHD (Kothari, 2012). Self-reported BN diagnosis was also predictive of inattention/hyperactivity in children at age 3 (Micali, Stahl, Treasure, & Simonoff, 2014). Recent research has shown Attention Deficit Disorders (ADD) to be predictive of substance abuse in adolescence and adulthood (Pingault et al., 2012; Urcelay & Dalley, 2012; Van Emmerik-van Oortmerssen et al., 2012; Yoshimasu et al., 2012) and eating disorders (Biederman et al., 2007; Yoshimasu et al., 2012), which in this study we found to be highly comorbid even at low frequency of purging. In the light of the increasing literature suggesting their association, it is perhaps possible to speculate on the existence of an underlying attention-deficit phenotype common to ADD, substance abuse and purging behaviours. More longitudinal research disentangling these associations is needed.

Our findings indicate the adolescents engaging in purging behaviours could more generally be considered as ‘at risk’ because of this clustering of risk behaviours. Public health initiatives focused on reducing risk behaviours among adolescents need to account for this high cooccurrence of behaviours and should be directed at ‘at risk’ adolescents engaging in any combination of risk behaviours. The impact of population-level strategies focusing on preventing risk behaviours among adolescents might also be enhanced by incorporating several risk behaviours, such as purging, substance use and depressive symptoms, rather than focusing on one behaviour at a time.

**Key Points**

- Purging disorder has been associated with psychological comorbidity; but most studies have focused on adult populations. There is some indication that purging behaviours might be more prevalent in mid- to late adolescence.
- The prevalence of purging behaviours was high; with cross-country variations suggesting a role for socio-cultural risk factors;
- Purging was associated with substance use and psychiatric comorbidity irrespectively of the frequency of purging and of binge eating.
- Purging behaviours are common among adolescent girls
- Girls showing purging behaviours are highly likely to have concurrent psychopathology and substance use.

## Supporting information

**Table S1** Baseline sociodemographic characteristic of all 16 year-old girls. **Table S2** Distribution of sociodemographic characteristics across girls. **Figure S1** Meta-analysis of the association between purging and binge drinking. **Figure S2** Meta-analysis of the association between purging and cigarette smoking. **Figure S3** Meta-analysis of the association between purging and drug use. **Figure S4** Meta-analysis of the association between purging and cannabis use. **Figure S5** Meta-analysis of the association between purging and depression.
