# Supplementary material for: Prevalence of purging at age 16 and associations with negative outcomes among girls in three community-based cohorts
Source: J Child Psychol Psychiatry. 2014 Jun 27;56(1):87–96. doi: 10.1111/jcpp.12283 (PMC4303957; doi:10.1111/jcpp.12283)
Supplement: Supplementary file 1 [file jcpp0056-0087-sd1.docx]

**Supplementary material**

**Table S1: Baseline socio-demographic characteristic of all 16 year old girls who were eligible to be sent the questionnaire at 16, of those who returned the questionnaire and those who had complete data on the variables included in the analyses**

|  | **ALSPAC^a^** | | | **GUTS^b^** | | | **NFBC^a^** | | |
| --- | --- | --- | --- | --- | --- | --- | --- | --- | --- |
|  | **Eligible sample** | **Returned question.** | **Complete cases** | **Full sample** | **Returned question.** | **Complete cases** | **Eligible sample** | **Returned question.** | **Complete cases** |
| **N sample** | **(N=4,462)** | **(N=2,742)** | **(N=1,608)** | **(N=9,039)** | **(n=4,915)** | **(n=3062)** | **(N=4,271)** | **(N=3,598)** | **(N=2,306)** |
|  | N(%) | N(%) | N(%) | N(%) | N(%) | N(%) | N(%) | N(%) | N(%) |
| **Maternal Marital Status** |  |  |  |  |  |  |  |  |  |
| *Married or cohabiting* | 3,501  (79.7%)  (n=4,392) | 2,224  (82.8%)  (n=2,711) | 1,347  (83.8%)  (n=1,608) | 7,284  (88.9%)  (n=8,194) | 4,103 (89.2%)  (n=4,599) | 2,724 (89.0%)  (n=3,062) | 4,036  (94.7%)  (n=4,263) | 3,420  (95.2%)  (n=3,591) | 2,190  (95.1%)  (n=2,302) |
| **Ethnicity** |  |  |  |  |  |  |  |  |  |
| *White* | 4,074  (95.9%)  (n=4,392) | 2,558  (96.5%)  (n=2,650) | 1,539  (96.9%)  (n=1,608) | 8,709  (96.4%)  (n=9,039) | 4,756 (96.8%)  (n=4,915) | 2,962 (96.7%)  (n=3,062) | N/A** | N/A** | N/A** |
| **Maternal education** |  |  |  |  |  |  |  |  |  |
| *GCSE+/Matriculation exam(NFBC)* | 1,716  (39.7%)  (n=4,324) | 1,236  (46.0%)  (n=2,685) | 787  (48.9%)  (n=1,608) | N/A* | N/A* | N/A* | 1,122  (30.1%)  (n=3,723) | 991  (31.4%)  (n=3,156) | 697  (34.0%)  (n=2,048) |
|  |  |  |  |  |  |  |  |  |  |

* Totality of sample: Nurses

** Totality of sample: White Caucasian

^a^ Baseline variables collected at birth – twins and triplets excluded

^b^ Baseline variables collected at age 9-14

**Table S2:** **Distribution of socio-demographic characteristics across girls reporting purging and those who did not in the 3 samples.**

|  | **ALSPAC (n=1,608)** | | | **GUTS (n=3,504)** | | | **NFBC (n=2,306)** | | |
| --- | --- | --- | --- | --- | --- | --- | --- | --- | --- |
|  | **Purging, N(%)** | **Non-purging, N(%)** | **p(χ^2^)** | **Purging, N(%)** | **Non-purging, N(%)** | **p(χ^2^)** | **Purging, N(%)** | **Non-purging, N(%)** | **p(χ^2^)** |
| **Child’s Ethnicity** |  |  |  |  |  |  |  |  |  |
| *White* | 149  (95.5) | 1,390  (97.1) | 0.3 | 195  (99.0%) | 3198  (96.7%) | 0.08 | N/A*** | N/A*** | **-** |
| *Non-white* | 7(4.5) | 42(2.9) |  | 2  (1.0%) | 109  (3.3%) |  | N/A*** | N/A*** |  |
| **Maternal education^*^** |  |  |  |  |  |  |  |  |  |
| *Up to O level(ALSPAC);*  *Comprehensive level (NFBC)* | 64  (40.7%) | 757  (52.2%) | 0.007 | N/A** | N/A** | - | 53  (65.4%) | 1,446  (65%) | 0.9 |
| *A level or more(ALSPAC);*  *Matriculation exam(NFBC)* | 93  (59.2%) | 694  (47.8%) |  | N/A** | N/A** |  | 28  (34.6%) | 779  (35%) |  |
| **Maternal marital status*** |  |  |  |  |  |  |  |  |  |
| *Single (single parent/divorced/widowed)* | 26  (16.6%) | 235  (16.2%) | 0.9 | 21  (10.7%) | 357  (10.8%) | 0.9 | 14  (17.3%) | 267  (12%) | 0.1 |
| *Married or cohabiting* | 131  (83.4%) | 1,216  (83.8%) |  | 176  (89.3) | 2,950  (89.2%) |  | 67(82.7%) | 1,958  (88%) |  |
|  |  |  |  |  |  |  |  |  |  |
|  | **ALSPAC** | | | **GUTS** | | | **NFBC** | | |
|  | **Purging, Mean(SD)** | **Non-purging,**  **Mean(SD)** | **F(p)** | **Purging, Mean(SD)** | **Non-purging, Mean(SD)** | **F(p)** | **Purging, Mean(SD)** | **Non-purging, Mean(SD)** | **F(p)** |
|  |  |  |  |  |  |  |  |  |  |
| **Age (years)** | 16.6(0.23) | 16.7(0.23) | 1.3(0.2) | 16.5(0.3) | 16.5(0.3) | 0.01 (0.9) | 15.3(0.6) | 15.2(0.5) | 0.75(0.4) |
| **BMI** | 22.3(3.6) | 21.6(3.5) | 6.3(0.01) | 22.1(3.0) | 21.6(3.3) | 3.22 (0.07) | 21.5(2.8) | 21.1(3.1) | 1.64(0.2) |

*In ALSPAC marital status is recorded at birth, in GUTS and NFBC at 16.

**Totality of sample: Nurses

*** Totality of sample: White Caucasian

**Figure S1: Meta-analysis of the association between purging and binge drinking**

**
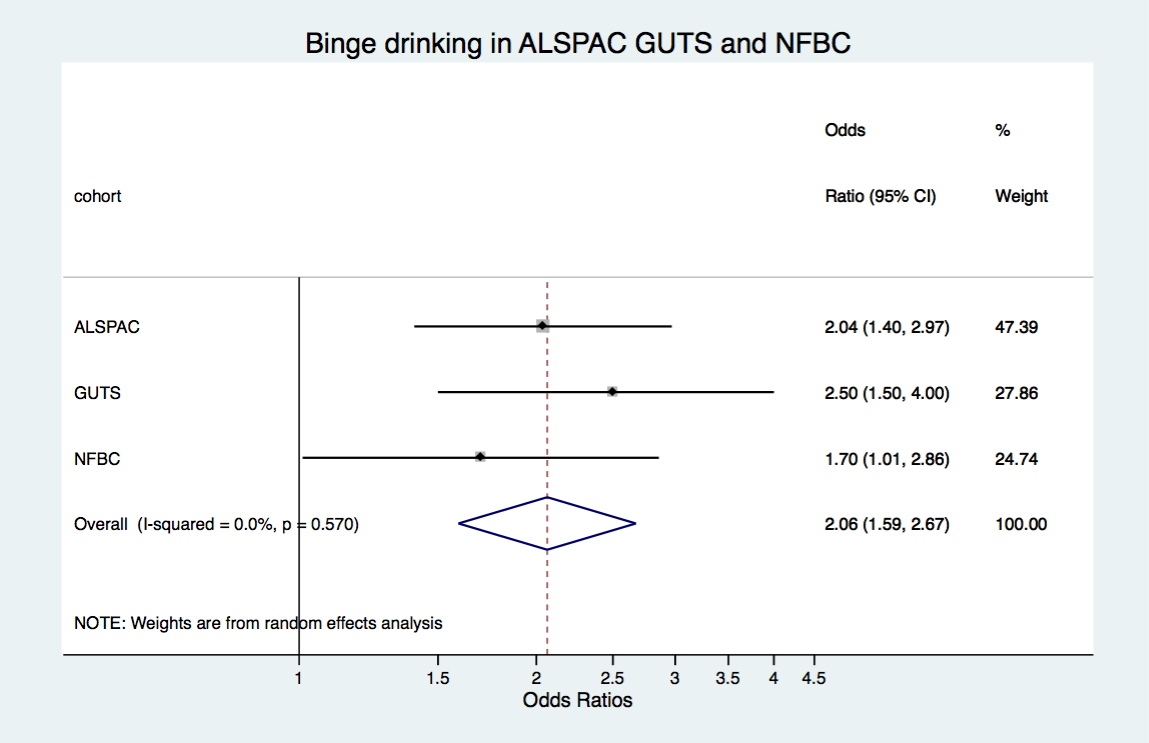
**

**Figure S2: Meta-analysis of the association between purging and cigarette smoking**

**
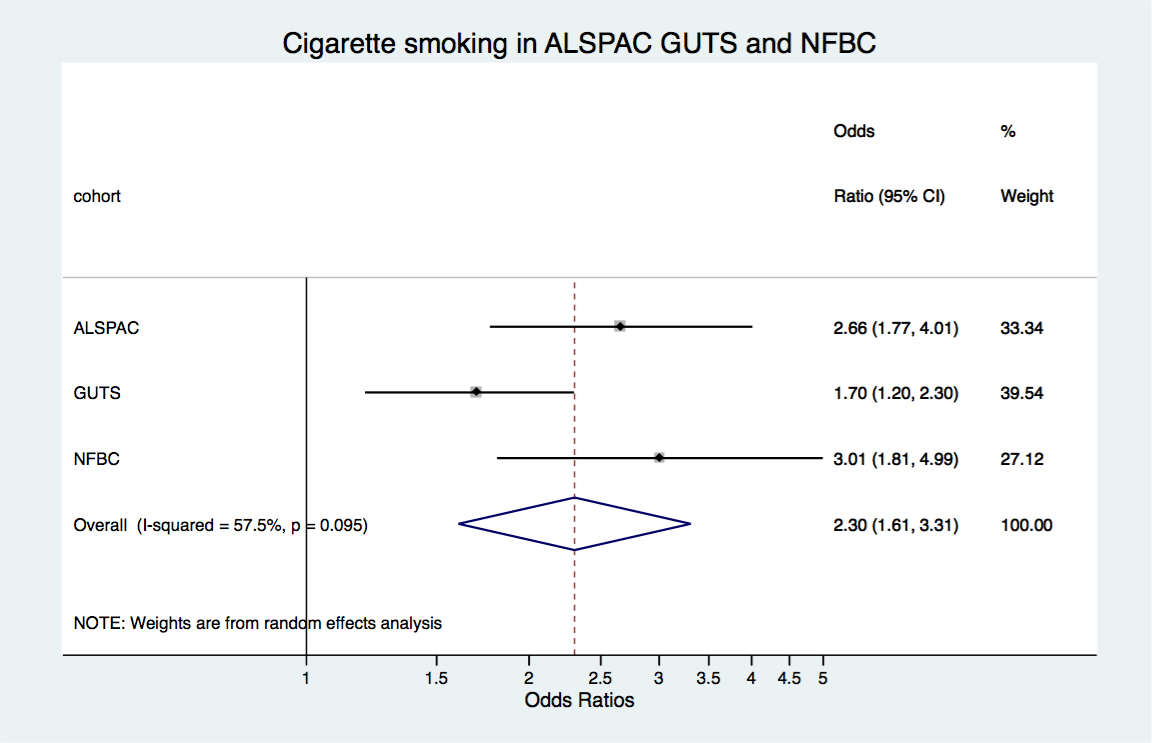
**

**Figure S3: Meta-analysis of the association between purging and drug use**

**
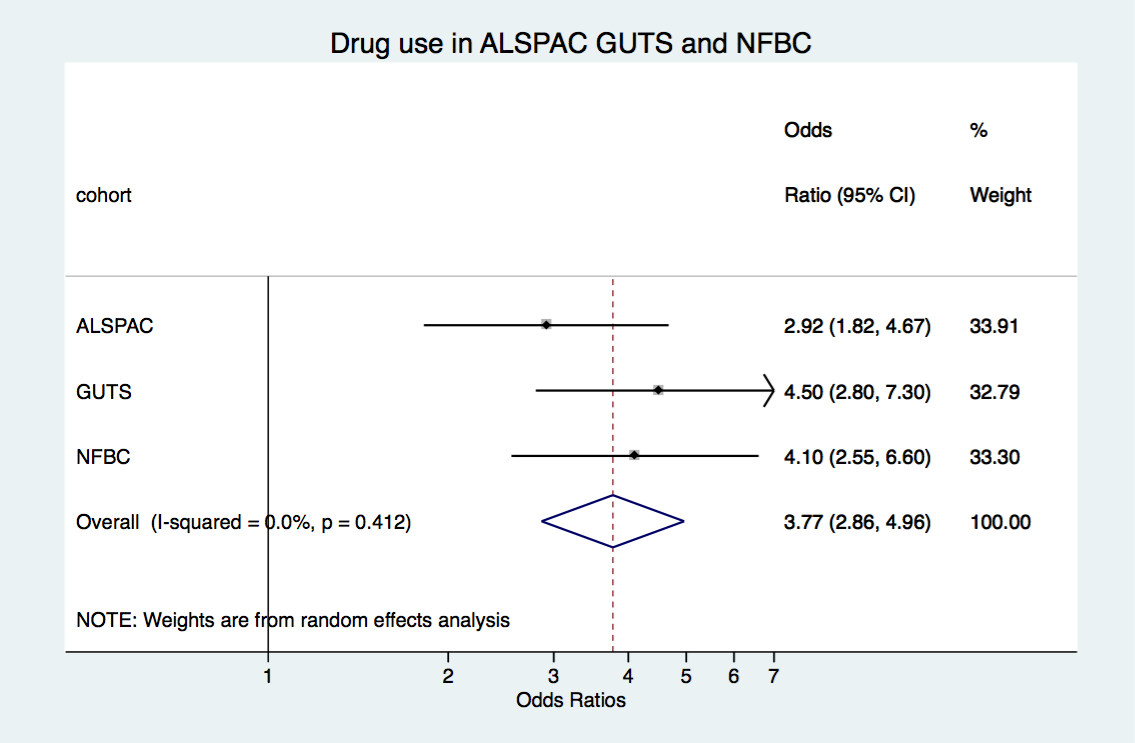
**

**Figure S4: Meta-analysis of the association between purging and cannabis use**

**
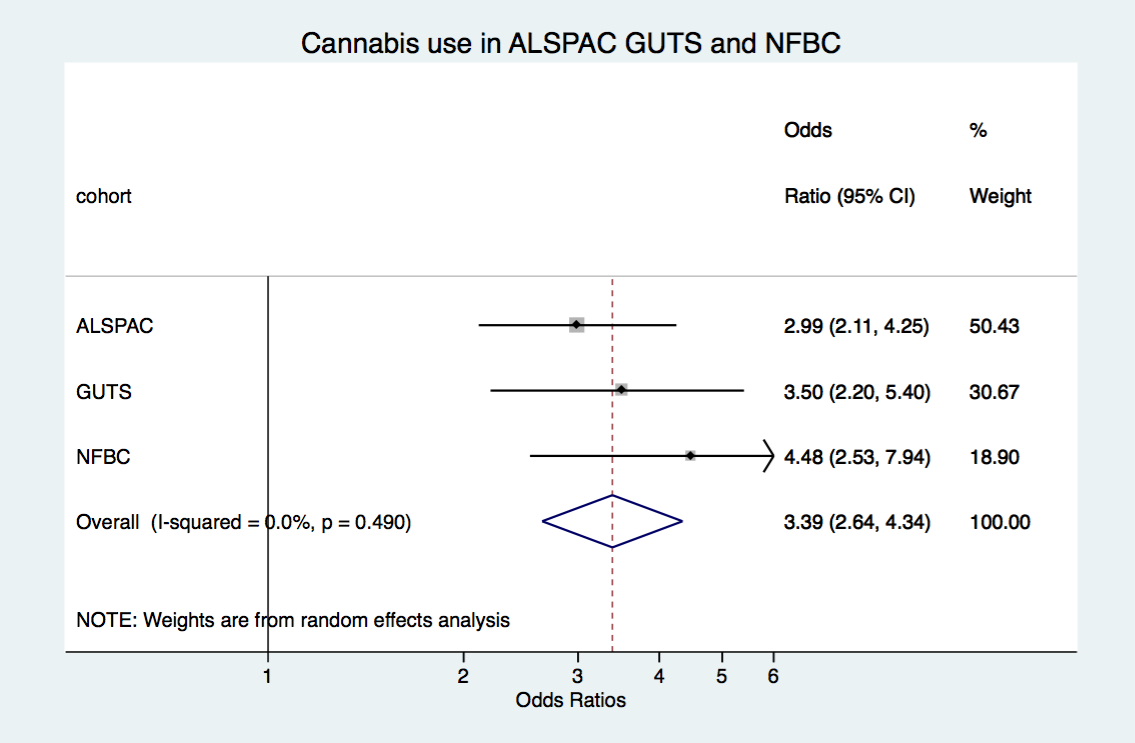
**

**Figure S5: Meta-analysis of the association between purging and depression**

**
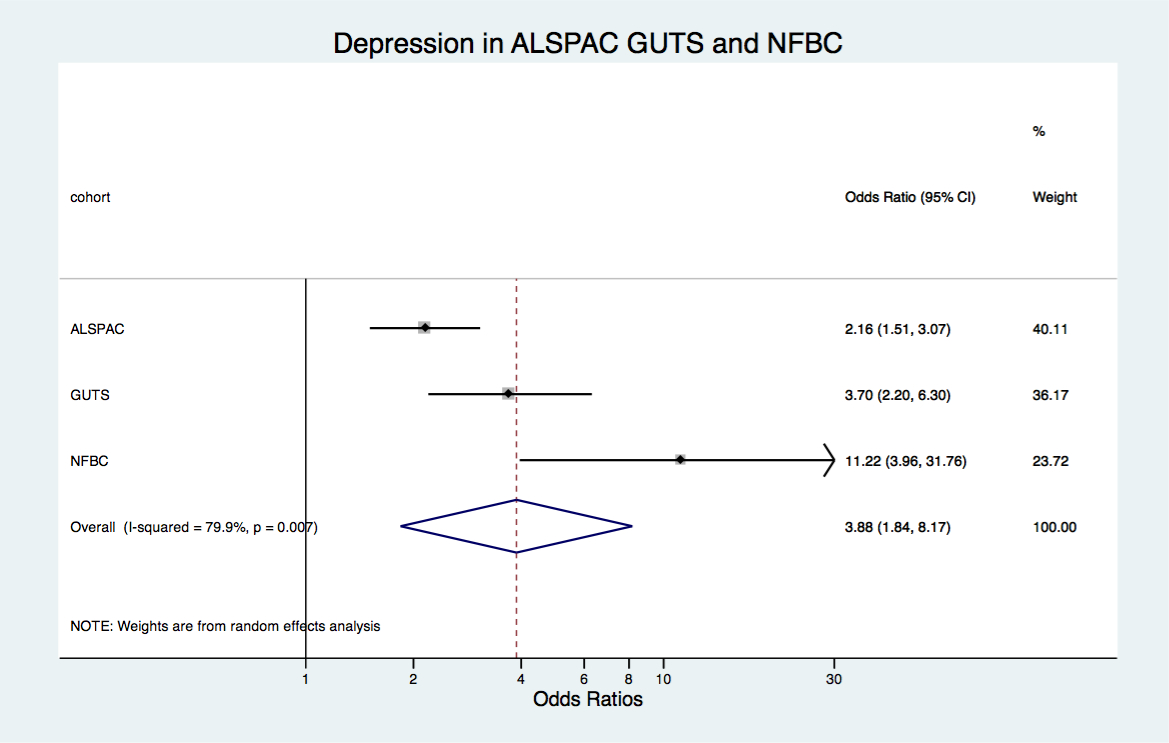
**
